# Blockchain Socket Factories with RMI-Enabled Framework for Fine-Grained Healthcare Applications

**DOI:** 10.3390/s22155833

**Published:** 2022-08-04

**Authors:** Saleem Ahmed, Abdullah Lakhan, Orawit Thinnukool, Pattaraporn Khuwuthyakorn

**Affiliations:** 1 Department of Computer System Engineering, Dawood University of Engineering and Technology, Karachi 74800, Sindh, Pakistan; 2 Department of Computer Science, Dawood University of Engineering and Technology, Karachi 74800, Sindh, Pakistan; 3 College of Arts Media and Technology, Chiang Mai University, Chiang Mai 50200, Thailand

**Keywords:** client–server, RMI, blockchain, socket, storage

## Abstract

The usage of digital and intelligent healthcare applications on mobile devices has grown progressively. These applications are generally distributed and access remote healthcare services on the user’s applications from different hospital sources. These applications are designed based on client–server architecture and different paradigms such as socket, remote procedure call, and remote method invocation (RMI). However, these existing paradigms do not offer a security mechanism for healthcare applications in distributed mobile-fog-cloud networks. This paper devises a blockchain-socket-RMI-based framework for fine-grained healthcare applications in the mobile-fog-cloud network. This study introduces a new open healthcare framework for applied research purposes and has blockchain-socket-RMI abstraction level classes for healthcare applications. The goal is to meet the security and deadline requirements of fine-grained healthcare tasks and minimize execution and data validation costs during processing applications in the system. This study introduces a partial proof of validation (PPoV) scheme that converts the workload into the hash and validates it among mobile, fog, and cloud nodes during offloading, execution, and storing data in the secure form. Simulation discussions illustrate that the proposed blockchain-socket-RMI minimizes the processing and blockchain costs and meets the security and deadline requirements of fine-grained healthcare tasks of applications as compared to existing frameworks in work.

## 1. Introduction

Innumerable emerging technologies will improve our health, including 6G wireless connection, blockchain technology, and programming interfaces of enterprise applications (such as sockets and remote method invocation) [1]. These technologies have been combined and designated as the new digital healthcare paradigm, composed of all the technologies mentioned earlier. The digital healthcare paradigm offers many remote healthcare services to mobile users to predict and analyze their healthcare issues 24/7. In practice, the users can diagnose many diseases at home by interacting with the hospital via different remote services. The latest mobile devices support many bio-healthcare sensors and enhance healthcare services for their users. Mobile devices exploit the Android operating system (X86) to run these applications. However, mobile devices suffer from resource-constrained issues and can not locally support these data-intensive and compute-intensive applications. One solution to this problem is that users can enhance the resources such as the battery, storage, and CPU inside mobile devices. However, this solution suffered from high processing and storage costs for mobile users [2]. Many solutions are suggested in the state-of-the-art to solve the resource-constraint issues of mobile devices. For instance, the remote procedure call (RPC) technique, remote method invocation (RMI), common object request broker (CORBA), and others. The goal is to offload the heavyweight workload from mobile devices to rich resource servers of mobile devices for execution. The RMI, CORBA, and RPC exploit the socket-based architecture where users and servers are separated, as in the client and server architecture. The client and server architectures are widely designed based on socket programming, where socket client and socket server classes are designed into the application programming interface [3,4,5]. Recently, the socket integrated cloud computing services to enhance the performance of the client–server model to support healthcare applications. The fog node is an extended version of cloud computing that allows services at the edges of the user network. The socket programming model enables mobile devices to offload their workloads to the fog and cloud computing for execution [6]. The remote procedure call (RPC) is widely exploited with the socket to run these healthcare applications in the mobile-fog-cloud network. Cloud computing offers different resources based on on-demand, on-reserve, and spot-instantaneous models. The socket implemented these resource models with RPC and executed the distributed healthcare applications on mobile-fog-cloud networks. Distributed healthcare applications are those in which workloads run on different nodes and store their data on other storage [7,8,9].

Challenges: In practice, the existing blockchain technologies such as Ethereum, Corda, Fabric, and IBM are widely exploited for healthcare applications. However, many research challenges exist in the current blockchain technologies and their approaches to RPC socket architectures [1,2,3,4,5,6,7,8] for healthcare applications. (i) The existing client–server architecture based on socket RPC suffers from high processing costs for cloud computing. (ii) The two-tier mobile and fog socket RPC frameworks suffer from resource balancing, processing costs, and storage costs for healthcare applications. (iii) Security is the key issue between socket layers in the existing RPC architecture for healthcare applications.

Contribution: This study devises the blockchain-RMI socket-enabled framework for fine-grained medical applications in mobile-fog-cloud paradigms. The goal is to minimize processing and storage costs and meet the security, privacy, deadline, and resource balancing constraints of work. This study considers one healthcare application consisting of different fine-grained tasks with deadlines and operations. This study finds the decentralized nodes (mobile, fog, and cloud) where each node can connect to another node based on blockchain rules to exchange the data based on security and privacy rules. In practice, the thin client mobile device only installs lightweight RMI interfaces, has a connection with the fog and is cloud-based on socket clients and socket servers. The data validation in terms of security will be evaluated based on blockchain technology, which converts the workload into a cipher based on AES-256 during processing in the system. The fog and cloud nodes offer processing and storage resources based on a serverless model where users pay for their usage instead of long-term provisioning of the cloud.

1 This paper designs the socket programming integrated remote method invocation (RMI) runtime interface based on Android X86 for healthcare applications in a blockchain-enabled mobile cloud network. The applications are distributed and run on different nodes with the same environment (X86) based on blockchain data validation. Generally, it is a blockchain-enabled RMI-socket enabled framework for healthcare applications in mobile-fog-cloud networks to minimize the processing cost and storage and meet all the given constraints.
2 This study drives the serverless processing cost model, which will charge based on execution time and is different from the existing hourly, weekly, and monthly on-demand services model. The goal is to minimize the processing cost for the healthcare application components and execute them within their deadlines.
3 This study invents the three-layer resource-balancing storage on mobile cloud computing, in which applications can be executed without the issue of scalability, reliability, and storage cost during processing. The mobile devices offload their workload to the fog nodes, and their results are offloaded to cloud computing with the minimum storage cost.
4 The data sharing and exchange from mobile devices and fog to cloud nodes for computing and storage has high security and privacy issues in mobile cloud computing paradigms. Anonymous external attacks and threats exist on the network, and healthcare-sensitive data could be compromised. This study devises the three-layer blockchain mining manager to create and add new blocks to the mobile-cloud network. Each block has its data hashing and nonces and validates each data transaction of the previous node in work.
5 This study presents the new simulator for healthcare applications based on an RMI-socket with a blockchain-enabled mobile-fog-cloud network to run distributed applications. For the data validation and hashing matching, this study devised the distributed hybrid offloading method to enable proof of work, ensuring security and privacy inside the framework for healthcare applications.

## 2. Related Work

This section discusses the related healthcare applications implemented based on client–server mechanisms in the fog cloud networks. In [1], a remote method invocation (RMI)-based healthcare framework was suggested for resource-constrained mobile devices to offload their data to cloud computing for further analysis. In practice, the work obtained optimal results and improved the quality of experience on the mobile device applications. The remote procedure call (RPC)-based framework was suggested for healthcare applications in [2]. The study triggers remote services when the mobile workload offloads data to remote servers for execution. It is an Android-X86 level service and runs at the operating system level when RPC is implemented at the kernel level inside the system. The study obtained the optimal objectives in terms of resource balancing and offloading in the considered problem.

Table 1 shows the related work of existing client–server architecture and their security mechanism based on existing security and blockchain in the distributed mobile edge cloud network. The main limitations with the RPC and RMI are live client and server connection stability, where intermittent service changes make the connection unreliable for the applications. The connection-enabled framework based on sockets is presented in [3,5]. The study achieved a strong connection and stored the running data on the cache without disruption of the request of applications during their executions.

Regardless, these sockets, RMI, and RPC did not support the security mechanism for healthcare applications in an open network. Security is a critical issue in an available network where many types of nodes are connected, and centralized security on one node cannot give feasible solutions to the applications. Decentralized and autonomous security mechanisms based on blockchain technology have been suggested in [4,6,7,8,9,10]. These blockchain-based frameworks improve the application-level abstractions of the Ethereum blockchain for healthcare applications. The applications offload their data into valid data transactions among all connected nodes.

These security mechanisms are based on extended versions of the existing security algorithms. For instance, AES-256, RSA, CRC-32, and others encrypt and decrypt data with asymmetric and symmetric paradigms for healthcare applications in the network. These algorithms are resource and time-hungry and are implemented in the blockchain technology to achieve hashing for security purposes only. The blockchain-enabled AES-256, SHA-256 and RSA-based solutions presented in [11,12,13,14,15,16,17] make valid and immutable transactions between connected autonomous nodes for healthcare applications. The RMI interfaces implemented for the blockchain classes, however, still consume much more of the server’s resources and lead to high processing costs for the applications.

The RPC-based blockchain presented in [18,19,20,21,22,23,24] to modify the blockchain technologies from bitcoin applications into healthcare applications. The RPC offers embedded level abstraction and allows modification inside the operating system to support healthcare applications based on blockchain technologies. These applications successfully obtained the security objectives and ran the healthcare applications on different nodes. However, applications’ delay, cost, and deadline are widely compared in these frameworks. The energy and delay level blockchain frameworks suggested in [25,26,27,28,29,30] improve the constraints on the above frameworks; however, resource consumption, costs, and deadlines of applications are still compared to the state-of-the-art blockchain in the system. The blockchain consensus has been widely implemented for different applications such as proof of work [31], proof of stake [32], delegated proof of stake (DPoS) [33], and leased proof of stake (LPoS) [34] to enable transaction validation in the blockchain nodes during processing in the network.

In the proposed work, we introduce novel blockchain socket factories with an RMI-enabled framework for fine-grained healthcare applications. The main objective of this study is to minimize processing and blockchain validation costs while meeting the application deadlines and security constraints in distributed mobile, fog, and cloud networks. The costs are determined by the processing scheduling cost, security encryption and decryption and validation cost, and storage cost for healthcare applications.

Table 2 determines the mathematical symbols of the problem and their descriptions.

## 3. Proposed Blockchain Enabled RMI-Socket Framework

**Figure 1:**
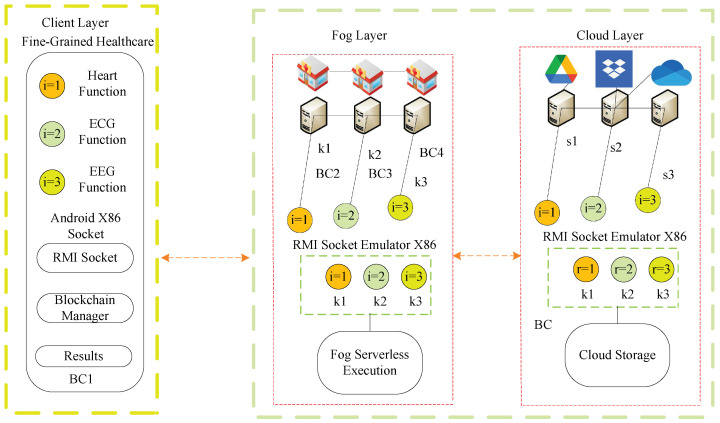
Blockchain RMI Socket-Enabled Framework.

This study devises the three-layer blockchain-enabled RMI-Socket framework, as shown in Figure 1. The client layer is the application, which consists of different fine-grained functions. Each function can be executed and offloaded with detailed data at the client layer. The procedures are only overridden methods from RMI-Socket, which offload data to the fog layer for execution. For instance, three functions, ECG, EEG, and numeric heart function, have their own data and deadline constraints. The client node can accept data as the request for the particular function and apply a hash on the request based on the blockchain and offload it to the fog layer for data analysis and execution. The offloaded hash data of all functions are to be validated and verified with the private key as the signature and the public key as the validation. All the fog nodes are homogeneous and can exchange their data for execution.

The cloud layer only offers storage services to avoid the storage costs of the fog nodes, which are not cost-efficient and are expensive in terms of saving data for a long time. All three layers were designed based on the RMI-Socket client, where the Java virtual machine (JVM) supports all Android X86 and Emulator X86 fog and cloud layers. All the nodes connect via socket-RMI registries in the framework.

### 3.1. System Model

**Figure 2:**
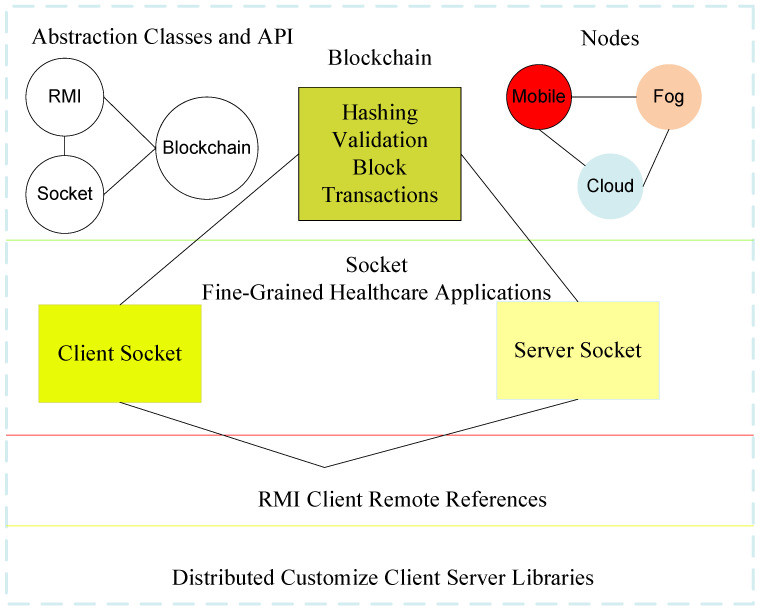
Abstraction of Socket RMI Blockchain for Healthcare Applications.

This study presents the designs for a blockchain-socket-RMI-enabled open-source framework for healthcare applications that consists of mobile, fog, and cloud nodes, as shown in Figure 2.

The proposed framework has an abstraction level where socket, RMI, and blockchain-customized libraries can be easily modified and updated for new healthcare applications in the network. The main goal is to develop such an application with only interfaces at the user level, implementation on the fog node, and storage of workload on the cloud-based blockchain technology. The blockchain is an open application programming interface (API) based on hashing, validation, and immutable transactions in the proposed integrated socket-RMI (client and servers) based on the interface level of RMI in the system. Therefore, it is a distributed and customized open-source framework that can be easily modified for new healthcare applications. Therefore, this study’s main goal is to suggest an open-source distributed socket-based client–server framework in which libraries can be updated for new applications in the network. The patients have mobile application access for uploading and downloading as well as doctor communication services in the system. The healthcare professionals have different hospital servers, which are implemented in different hospitals as the healthcare fog and cloud servers. Patients can update, upload, and download data from the mobile application’s patient access, and the healthcare professional can update, analyze, and classify the data on the server at different hospitals.

### 3.2. Node Scheduling

There are three nodes in the system, i.e., mobile node, fog node and cloud node, for healthcare application processing. The mobile node can only execute the lightweight tasks and offload them to the fog nodes for further processing with the minimum end-to-end latency, whereas fog nodes have minimum lateness due to the proximity of the site to the execution of the application. However, fog nodes have a higher storage cost for saving the data in the system. Therefore, we implement a public cloud node with fewer storage costs in the system. In general terms, the cloud has a longer communication delay but smaller processing and storage costs in the system. Therefore, to keep the balance between resource constraints, processing delay, and storage costs, this study implemented different nodes and ran the application on the mobile, fog, and cloud nodes in the system.

### 3.3. Security and Privacy Mechanism

In the system, we consider both the security and privacy mechanisms for the healthcare application. Privacy is the authenticated login and access of the controls in the client–server-based application, and security is the data validation, encryption and decryption and attacks in the system. We devised a blockchain-based security mechanism in which both security and privacy are maintained for the healthcare application in the system.

### 3.4. Problem Formulation

This study considers the healthcare application *I* with different fine-grained functions, e.g., {i=1,⋯,I}. Each fine-grained function *i* has workload $w_{i}$, deadline $d_{i}$ and storage address $s_{i}$ in the framework. All the fine-grained functions operate in real-time and are independent of each other in the framework. This study considers *K* number of serverless homogeneous fog nodes, e.g., {k=1,⋯,K} where each fog *k* has uniform speed $\zeta_{k}$ and resource $\epsilon_{k}$. This study considers the *S* number of heterogeneous cloud storage services, {s=1,⋯,S}, with the storage capacity, $\epsilon_{s}$. This study considers the *m* number of mobile devices, e.g., {m=1,⋯,M}, with the storage capacity $\epsilon_{m}$ and processing speed $\zeta_{m}$. Each mobile, fog and cloud has different blockchain blocks with the following attributes. For instance, a number of blocks, e.g., {BC=1⋯,BC} has BC=<hashing,private−key,public−key,pre−hash,timestamp> attributes.

(1) $$x_{i\in I}=\left\{\begin{array}{cc} \frac{w_{i}}{\zeta_{m}}, & x_{i}=1,\;mobile-assignment,\\ \frac{w_{i}}{\zeta_{k}}, & x_{i}=2,\;fog-assignment,\\ \frac{w_{i}}{\zeta_{s}}, & x_{i}=3,\;cloud-assignment. \end{array}\right.$$

Equation (1) determines the assignment of the fine-grained function on different mobile-fog-cloud networks. The fine-grained workload offload and blockchain validation time are determined in the following way. This is the binary assignment variable in the formulation.

(2) $$local_{i}^{e}=\sum_{i=1}^{I}\sum_{m=1}^{M}\frac{w_{i}}{\zeta_{m}}+BC_{1}+\tau_{i}.$$

Equation (2) determines the local execution and blockchain process at the local device during offloading in the framework. All blockchain attributes and validation on mobile devices are determined based on Equation (2)

(3) $$\begin{array}{c} BC_{i}=i\leftarrow <hashing,private-key,\\ public-key,pre-hash,timestamp>. \end{array}$$

Equation (3) determines blockchain offloading by requesting hashing and exchanging data to the fog cloud based on hashing, public key, private key, and timestamp.

(4) $$mobile_{i}^{cost}=\sum_{i=1}^{I}\sum_{m=1}^{M}local_{i}^{e}\times \phi_{i}.$$

Equation (4) determines local execution and offloading costs for the mobile devices.

(5) $$\tau_{i}^{m,k}=\frac{w_{i}}{Bandwidth}+logn\left(\frac{N}{noise}\right).$$

Equation (5) determines local execution time from mobile device to fog node based on exchanging data. The execution time on serverless fog nodes is determined in the following way.

(6) $$fog_{i}^{e}=\sum_{i=1}^{I}\sum_{k=1}^{K}\frac{w_{i}}{\zeta_{k}}+BC_{1}+\tau_{c}^{\prime }.$$

Equation (6) determines the execution on the fog nodes. The execution cost on serverless fog nodes is determined in the following way.

(7) $$fog_{i}^{cost}=\sum_{i=1}^{I}\sum_{k=1}^{K}\frac{w_{i}}{\zeta_{k}}+BC_{1}+\tau_{c}^{\prime }.$$

Equation (7) determines the execution cost of the fog nodes.

(8) $${\tau^{\prime }}_{i}^{k,c}=\frac{w_{i}}{Bandwidth}+logn\left(\frac{N}{noise}\right).$$

Equation (8) determines the exchange and offload of data between the fog and cloud.

(9) $$cloud_{i}^{cost}=\sum_{i=1}^{I}\sum_{s=1}^{S}\frac{w_{i}}{\zeta_{c}}+BC_{1}$$

Equation (9) determines the cloud storage cost in the framework.

The objective function of this study is to minimize the local cost, processing cost and storage cost of all fine-grained functions, and these can be determined in the following way.

(10) $$T_{total-Cost}=mobile_{i}^{cost}+fog_{i}^{cost}+cloud_{i}^{cost}.$$

Equation (10) determines the total cost of all fine-grained functions and their workloads.

## 4. Blockchain-Socket-RPC Algorithm Framework

This study devises the blockchain-socket-RMI for fine-grained healthcare applications in mobile-fog-cloud networks. The main objective is to design a flexible and reliable healthcare framework in which the total costs of fine-grained applications could be minimized. This study devises blockchain-socket-RMI schemes in which different phases are analyzed to meet the requirements of the fine-grained application, as shown in Algorithm 1.
**Algorithm 1**. Blockchain Socket RPC Algorithm Framework.
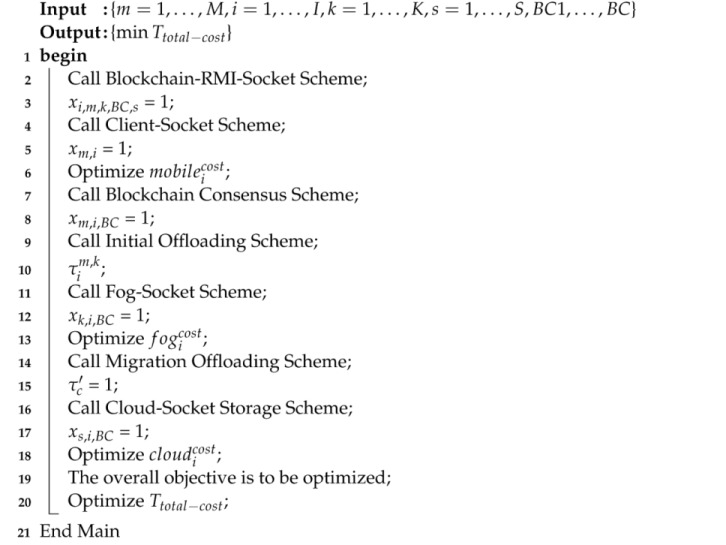


The proposed blockchain-socket-RMI consists of different schemes, as shown in Algorithm 1. The proposed algorithm determines the optimal allocation of all mobile, fog, and cloud costs for the healthcare function to work. All the schemes of Algorithm 1 solve the problem with the help of different methods explained in the following way.

- Client-Socket Scheme: In this scheme, we start the application process of the mobile devices, such as installed applications, and display the healthcare interfaces as fine-grained tasks. These tasks are fine-grained and have autonomous data for processing. Each workload encrypts and decrypts and is validated based on Equation (3) before offloading to the fog node for processing.
- Call Blockchain Consensus Scheme: In order to validate the fine-grained workload of healthcare data, we devise the partial proof of validation (PPoV) scheme at the mobile, fog and cloud nodes for validation during data migration.
- Initial Offloading Scheme: This scheme will allow the data to be encrypted and validated based on PPoV, and the data can be offloaded to the fog node for further processing.
- Fog-Socket Scheme: This is a scheduler where all requested fine-grained workloads are scheduled based on their given deadlines and cost constraints.
- Call Migration Offloading Scheme: This scheme offloads executed data to cloud computing to further analysis and storage in the framework.

Algorithm 2 validated each transaction between the mobile device and the fog node for each workload based on hashing values in the system. The data validation will be performed based on PPoV between the fog node and cloud node during data offloading in the system.
**Algorithm 2**. Partial proof of validation (PPoV).
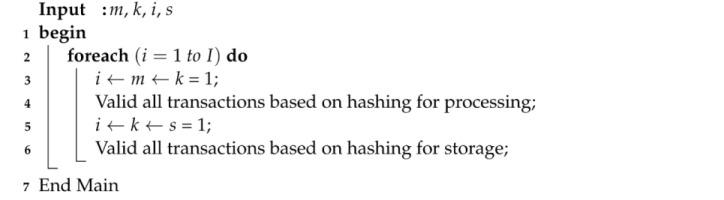


### 4.1. RMI-Socket-Registry

This study uses remote method invocation (RMI) to construct bespoke socket factories. Custom socket factories can be used to regulate how network-level remote method invocations are conveyed. They can be used to regulate socket settings, address binding, and data migration-based blockchain hashing and consensus methods. The framework creates the RMI registry at different nodes to ensure the network communication at different nodes and allow a serverless model to work with the mobile and cloud layers from the centralized fog layer. This study merged the factory pattern stub and skeleton with the client socket, server socket-based unicast binding, and acceptance rules in the three-layer socket. The data are exchanged in the network in the form of hashing instead of serialization. The RMI is the method in which three layers can exchange their data, and the socket offers the application programming interface and allows the method to declare and execute in different client–server nodes.

### 4.2. Blockchain Consensus of Mobile-Fog-Cloud-Socket-RMI Mechanism

**Figure 3:**
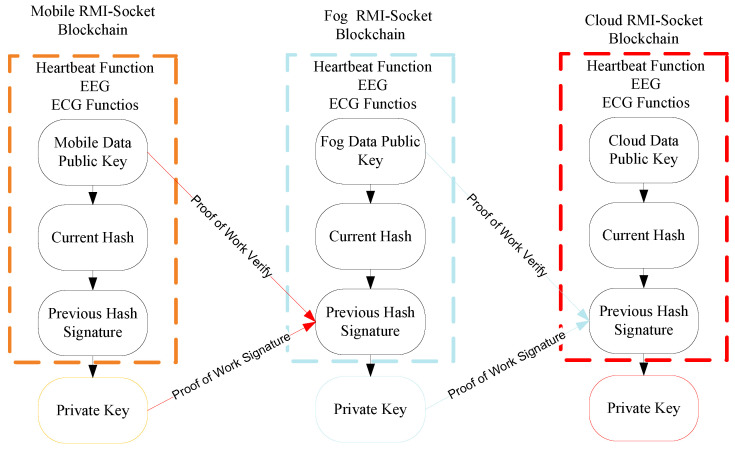
Blockchain RMI-Socket.

This study devises the proof of work with the signature matching and hashing validation features on the mobile, fog and cloud layers to ensure the security of the exchange of data between nodes, as shown in Figure 3.

The goal is to ensure security and privacy and restrict unauthorized access to the data in the distributed mobile-fog-cloud network. The fine-grained function data are to be converted into hash based on the designed public key and signature understanding based on the private key. The partial proof of validation (PPoV) method is distributed, ensuring both signature and validation on all interconnected nodes in the system. Figure 3 shows that all the exchange data are replicated on all nodes. However, the cloud only stores the processed data on different storage services.

### 4.3. Hybrid Offloading in Blockchain RMI-Socket

This study considers the hybrid offloading mechanism, which aims to minimize the processing cost and storage cost in the work. Initially, the fine-grained functions offload their data to the fog nodes for execution to minimize the processing cost of healthcare applications, where, after the execution, the fog nodes offload their application-related data to the cloud node for storage. In the system, there are two types of offloading performed between mobile and fog nodes and fog nodes and cloud computing.

Due to lightweight and constraint issues, the mobile devices offload their workloads to the available fog nodes, while after the execution of workloads at the fog nodes, the executed workload results are offloaded to the cloud computing for storage in the system.

### 4.4. Socket Offloading and Scheduling Scheme

In the proposed framework, three different computing nodes are implemented to facilitate users at their devices, create a robust and efficient execution with minimum processing time and cost, and provide low-cost storage for the healthcare application. This study presents a hybrid offloading and scheduling-enabled cost-efficient scheme that executes all fine-grained tasks with deadlines and validates the data transactions in a secure form with minimum system costs. The algorithm has two ways of offloading: offloading data between mobile devices to the fog node and fog node to cloud computing for a single application in the network. The scheduler allocates applications on the three different nodes during execution. Therefore, executing the applications among resource constraint devices and rich resource servers with optimal results is more reasonable. This study devises the cost-optimal mobile-fog-cloud offloading and scheduling scheme to ensure the execution of fine-grained tasks is based on their requirements.

Algorithm 3 determines the cost-efficient scheduling and offloading in the mobile-fog-cloud network for the fine-grained healthcare functions of the application. From steps 1 to 7, This study performs mobile computing on the blockchain with function requests at the minimum execution cost time and offloads them based on a proof of work method to ensure the signature and validation of the next fog socket. The initial offloading happened between the mobile-socket and fog-socket-based blockchain proof of work. From steps 10 to 15, the fog nodes schedule all fine-grained functions on the serverless fog nodes and send their results to cloud computing for storage. All the function executions must be less than the capacity of the system’s mobile devices and fog nodes. Further offloading to be completed between the fog nodes and cloud computing for data storage based on the objective function is shown in steps 16 to 21.
**Algorithm 3**. Optimal cost mobile-fog-cloud offloading and scheduling scheme.
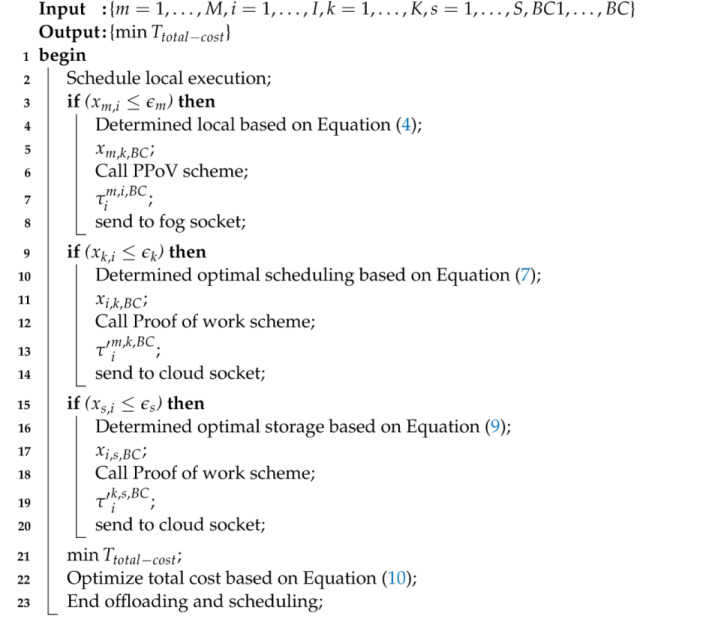


## 5. Performance Evaluation

In this part, we analyze the results, show the implementation, and evaluate the performances of schemes for the healthcare application. In the performance evaluation, we conducted experiments on different parameters, as defined in Table 3. The parameters are fine-grained tasks with their data, e.g., {i=1,⋯,I}, computing nodes {k=1,⋯,K} and users devices, m∼M. All the parameters are configured in the simulation file during the experiments for healthcare applications. The simulation was conducted on an open-source solidity framework along with the Android X86 Flutter emulator to ensure practical use of the applied engineering application. The main difference is that we only created the different interfaces at the client socket based on RMI, called and executed them on the fog nodes, and stored the application data on the cloud. We defined the implementation of the simulator in the respective subsection with the different abstractions of classes and methods.

Table 4 shows the different node costs in the simulation.

### 5.1. Implementation of Socket-RMI-Blockchain

**Figure 4:**
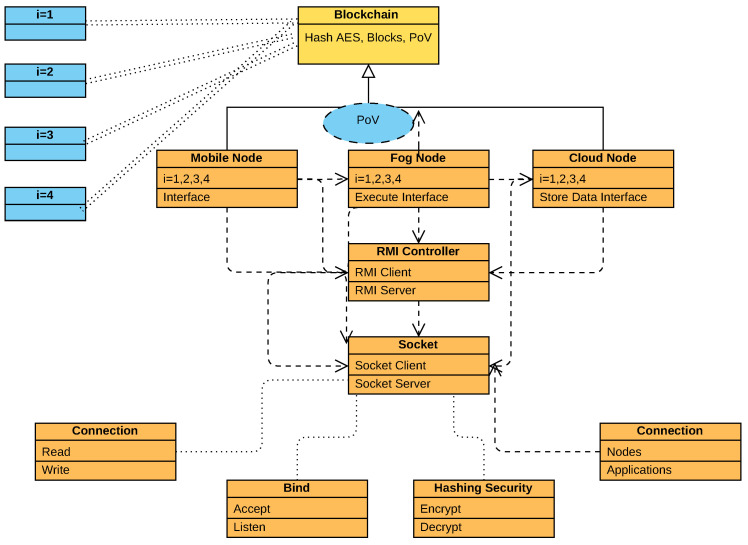
Abstraction of Socket-RMI-Blockchain for Healthcare Application.

This study defines the different classes from higher abstraction levels to integrated enumerated interfaces in the system, as shown in Figure 4.

The proposed framework has different abstractions of classes, such as the application of fine-grained tasks (e.g., *i* = 1, 2, 3, 4). The blockchain has four functions: hashing based on AES-256 (e.g., asymmetric mode), proof of validation, transaction of data, and block of unique numbers in the class. The partial proof of validation (PoV or PPoV) has to be used in different classes, such as mobile classes, fog classes, and cloud classes, for the validation of the data during their declaration, initialization, and calling in the framework. The mobile node only shows the interfaces for the healthcare functions, such as heart function, ECG function, EEG function, and general doctor–patient monitoring tasks. However, the data are encrypted and decrypted at the mobile devices and offloaded to the fog node for further processing. The fog node has two objectives. At first, the fog node validated the offloaded interface data based on the PPoV scheme and applied execution on the interfaces. The fog nodes offloaded the processed data to the cloud for the storage interface at work. The RMI controller is the main class, which consists of many interfaces such as client, server, connection, acceptance, and others. The RMI controller classes implement the interfaces inside the socket. At the same time, the socket is the open-source API that consists of a client stub socket and a server skeleton socket with the different sub-classes. These classes are connection (e.g., read/write properties), bind (e.g., accept and listen), hash security transport level (encryption and decryption and validation), and client connection with applications and their nodes for the healthcare execution in the network.

In this work, we implemented the blockchain technology with the socket-RMI in the solidity framework with an open API for developers. The fundamental API guideline has been taken from the tutorial in [35], where all open-source classes are defined, and source code is available for further usage. Initially, the solidity framework implemented different smart-contract methods for the other blockchain frameworks, and for this, we have also modified the classes in the network in the current work.

### 5.2. Results Discussion

The execution cost is a logical cost of workload execution in a socket-based RPC framework. Socket programming has different steps, such as client-socket and server socket. The study implemented remote procedure call services with the designed blockchain technology in the implementation part. This study implemented the existing baseline approaches, such as blockchain-offloading [12,17,19,23,24], blockchain-socket [9,11,14,16,20] and proposed blockchain socket-RPC, in the system.

Socket programming has a peer-to-peer network in terms of client–server architecture; in RPC, we considered the different nodes, and each workload was offloaded to the one fog node. The blockchain process was performed on the same machine as the initial execution and with the minimum hashing and proof of work validation in the blocks. All the workloads are hashed, based on SHA-256, and make transactions between the mobile device client socket and the server socket fog node for processing. The consensus algorithm validated each transaction using proof of work from execution to storage. The existing Ethereum [11] and Fabric [12] blockchains for distributed healthcare applications have a lot of validity and security. However, Ethereum and Fabric were initially designed for financial applications and, thus, need a lot of resources for execution.

**Figure 5:**
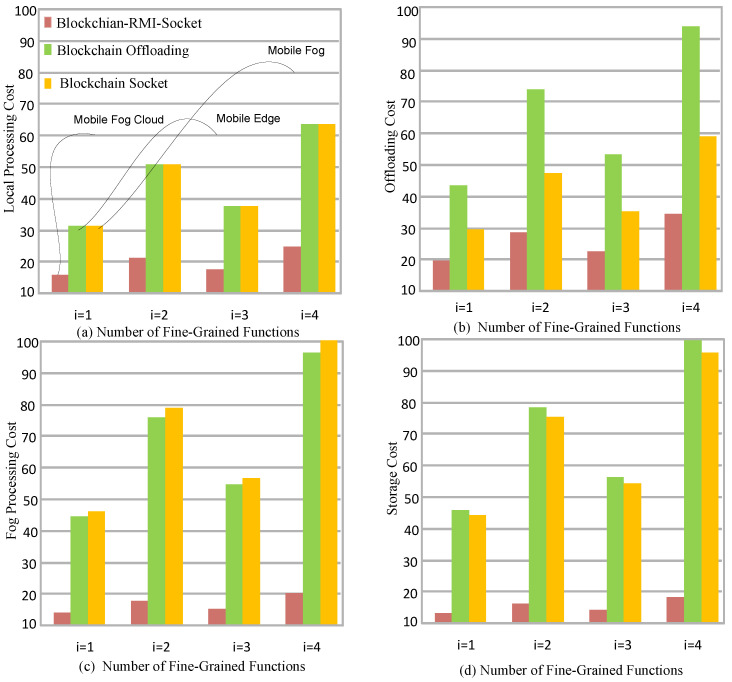
Cost-Efficient RMI-Socket-Blockchain System for Healthcare Functions in a Mobile-Fog-Cloud Network.

Figure 5a shows the local cost execution during the initial phase of the blockchain process and then offloads it to the fog node for execution. The signature and validation process of proof of work is applied at each node; therefore, each process has a cost at each node. Figure 5a shows that the proposed RMI-socket blockchain gained optimal results compared to the existing offloading blockchain and socket-blockchain. The main reason is that all of the existing blockchain frameworks only focus on security and validation and ignore the offloading cost and processing in their model. However, this study devises the lightweight proof of work based on serverless functions and has less resource consumption than existing proof of work and validation in the mobile-fog-cloud environment. The proposed system has efficient layers in which mobile applications offload secure data to the fog node for processing, and fog nodes save their results on cloud computing with the cheaper data storage services in the system. Figure 5b,c shows that the blockchain cost and fog and cloud cost were the optimal systems in the proposed work compared to existing blockchain-based systems for fine-grained applications.

Figure 5 shows that the proposed blockchain RMI-socket is more efficient in terms of cost compared to the existing one. Each hospital offers different storage options in the framework, as shown in Table 4. Figure 5d shows that the optimal storage from an existing third-party provider suffered from resource leakage due to the limited resource availability in their fog nodes.

Figure 5d shows that the storage mechanism of the proposed blockchain socket RPC has fewer service costs compared to the existing storage and processing costs of socket-based methods and RPC-based methods for healthcare applications. The main goal of this study was to divide the workload between different nodes as existing workloads were executed on the same node, and the execution of this huge workload results in extra costs. Then, the workload is offloaded to a particular hospital, which has different fog nodes based on their costs. All workloads were executed on schedule.

**Figure 6:**
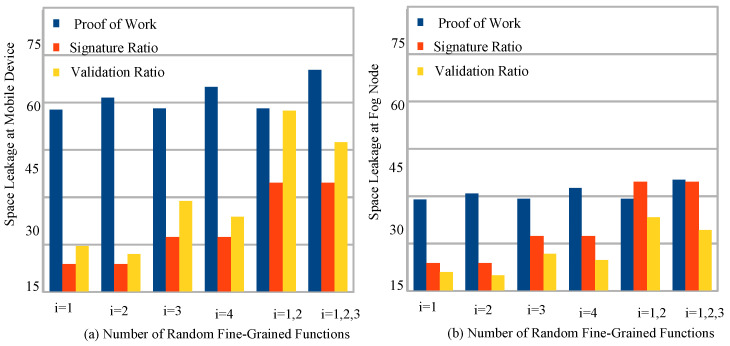
Resource Leakage Issue in Blockchain Technologies While Performing Cryptograhic Schemes for Healthcare Applications.

Figure 6a,b shows the resource leakage at the mobile devices and fog nodes during the implementation of blockchain technology with the proof of work, signature matching and node and hash validation in the two-node network. Figure 6a shows that mobile devices have higher resource leakage because of their limited resource capacity and can not locally support the entire blockchain mining process for fine-grained functions. Figure 6b shows that the fog nodes cannot support the entire blockchain process during execution and storage during the random arrival of hash data to the system. The main reason is that all the blockchain cryptographic schemes are resource-hungry and require a lot of resources to meet their validation goals for healthcare applications. Therefore, there are many possibilities that it may face resource leakage during the blockchain validation for the healthcare application with the different schemes for the specific node. Therefore, this study devised a three-layer lightweight blockchain mechanism where fine-grained functions are executed on different nodes to manage their resource leakage and minimize the processing and storage costs of the system.

### 5.3. Proof of Validation of Fine-Grained Tasks

This study compared the consensus methods of blockchain technologies for healthcare applications in a simulation environment. This study implemented four existing blockchain consensus schemes, i.e., PoW [31], PoS [32], DPoS [33] and LPoS [34], to compare them with the PPoV for fine-grained healthcare applications in the framework.

**Figure 7:**
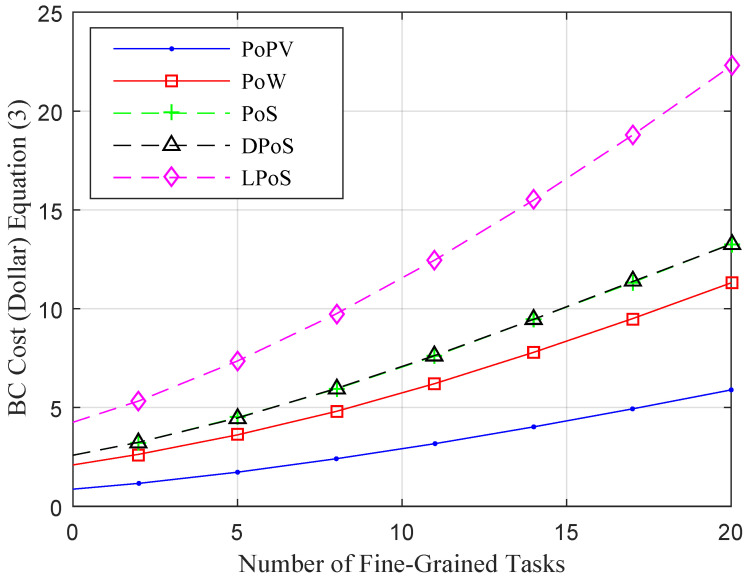
Proof of Validation Costs with All Healthcare Application Fine-Grained Tasks.

Figure 7 shows that the proposed PPoV has lower processing costs compared to all consensus schemes when running healthcare applications in a mobile-fog-cloud environment.

Initially, in the simulation environment, we submitted all fine-grained tasks to the system and analyzed the cost of application during execution in the system. After that, we submitted random fine-grained tasks to the system for execution. Figure 7 shows that partial validation on different nodes is lightweight and has lower processing and validation costs than existing blockchain methods during the processing of an application in the system.

The blue PPoV line shows the lowest processing costs for fine-grained tasks of an application in the system.

Compared to other consensus blockchain algorithms, the PPoV outperformed in terms of cost and security validation among nodes in the system. There are many reasons for this better performance. (1) All the consensus blockchain algorithms work on homogeneous nodes in the form chain and share common protocols during their data transactions. However, these algorithms only focused on security and data validation without checking the resource availability at the nodes for the verification during data transportation in the network. In the blockchain, each node must verify the data validation to make the transaction immutable. Therefore, each node requires substantial resources to make huge transactions for different fine-grained tasks. In the experiment section, we analyzed that heterogeneous nodes have higher processing costs that the existing consensus algorithms because not all nodes have the same computing capabilities, such as mobile, fog, and cloud; therefore, they lead to increased processing costs during transactions in heterogeneous nodes of healthcare applications. In our case, we checked the resources in advance and applied partial validation to avoid resource leakages and overflow at the nodes. For example, the mobile device can encrypt, decrypt, and offload data to the fog nodes; if the mobile device has sufficient resources, it may validate the data. Otherwise, fog nodes validate the data instead of the mobile device, and the mobile device then acknowledges the valid data, resulting in the lowest processing costs in the system. (2) Another reason is that we divided data validation among different nodes; the powerful nodes can validate the hashing of data efficiently compared to resource-constraint devices in the network. Hence, it has been proved that PPoV is a reliable and cost consensus method for fine-grained applications in a heterogeneous network and obtained the optimal results, as shown in Figure 7.

## 6. Conclusions

This paper presented the blockchain-socket-RMI-based framework for fine-grained healthcare applications in a mobile-fog-cloud network. The proposed partial proof of validation (PPoV) scheme outperformed all existing blockchain schemes and validated the data with the minimum processing costs compared to all blockchain consensus methods. This study presented the abstraction levels of classes in a framework, which can be further improved for other healthcare applications.

In our future work, we will optimize the energy efficiency of mobile devices, fog nodes and blockchain processes in the framework for healthcare applications.

## Figures and Tables

**Table 1:** Existing Healthcare Client Server Frameworks Based on RMI, RPC, and Socket.

Study	Hashing Techniques	Application	Architecture	Layers	Language	Node
[1]	MD5	Heartbeat	CORBA	Client–Server	JAVA	Mobile-Cloud
[2]	SHA-256	Blood-P	RPC	Client–Server	JAVA	Mobile-Cloud
[3]	AES	Healthcare	RPC	Client–Server	JAVA	Mobile-Cloud
[4,5]	RSA	Medical Care	RMI	Client–Server	C/C++	Mobile-Cloud
[6]	AES	Medical Care	SOA	Client–Server	C/C++	Mobile-Edge
[7,8,9,10]	Blockchain	Medical Care	Ethereum	Client–Server	PYTHON	Mobile-Edge
[11,12,13,14,15,16,17,18,19,20]	Blockchain	Medical Care	Open-Source	Client–Server	PYTHON	Mobile-Edge
[21,22]	Privacy	Healthcare	fixed	Client-client	PYTHON	Mobile-Edge
[23,24,25]	Privacy	Healthcare	fixed	Server-Server	PYTHON	Mobile-Edge
[26,27]	Privacy	Healthcare	fixed	Server-Server	PYTHON	Mobile-Edge
[28,29]	Privacy	Healthcare	fixed	Nodes	PYTHON	Mobile-Edge
[30]	Privacy	Healthcare	fixed	Hybrid-Client–Server	PYTHON	Mobile-Edge
Proposed	AES-256	Fine-Grained Tasks	RMI-Socket-Blockchain	Many Clients-Servers	JAVA	Mobile-Fog-Cloud

**Table 2:** Problem Constraints and Notations.

Notations	Description
*I*	Number of fine-grained healthcare functions
*i*	Fine-grained function *I*
*W*	Amount of function data
$w_{i}$	Particular data of function *i*
$d_{i}$	Deadline of fine-grained function *i*
*M*	Number of client nodes
*m*	Particular node such as mobile
$\epsilon_{m}$	Resources of particular node
$\zeta_{m}$	Speed of node *m*
*K*	Number of homogeneous fog nodes
*k*	Particular node such as fog node *k*
$\epsilon_{k}$	Resources of particular node
$\zeta_{c}$	Cloud storage processing node
$\zeta_{k}$	Speed of node *k*
BC	Number of blockchain blocks
Hash	Hash of the block
Pre-hash	Pre-Hash of the block
Private-Key	Private key of the block
Public-Key	Public key of the block
*S*	Number of cloud storage available
*s*	Particular storage of cloud
$logn(\frac{N}{noise})$	Logarithm of inference *N* and network noise
Bandwidth	Available bandwidth network

**Table 3:** Simulation Parameters of Blockchain Socket RMI for Fine-Grained Healthcare Application.

Config Parameters	Parameters Values
Socket-Programming API	JAVA
*i* = 1	200 MB heartbeat workload
*i* = 2	900 MB Blood pressure
*i* = 3	2 GB EEG Values
*i* = 4	4 GB MB ECG pictures
*i* = 5	600 MB heartbeat workload
*i* = 6	900 MB Blood pressure
*i* = 7	2 GB EEG Values
*i* = 8	4 GB MB ECG pictures
*i* = 9	1200 MB heartbeat workload
*i* = 10∼15	1900 MB Blood pressure
*i* = 16	5 GB EEG Values
*i* = 17∼20	7 GB MB ECG pictures
*m* = 1	Android 64 GB ROM, 8 GB RAM
*m* = 2	Android 128 GB ROM, 16 GB RAM
*k* = 1	Core I5 30 GB ROM
*k* = 2	Core I7 100 GB ROM
*k* = 3	Core I9 500 GB ROM

**Table 4:** Cost of Nodes.

Node	Cost
*s* = 1	2 dollar per Hourly use for applications
*s* = 2	3 dollar per Hourly use for applications
*s* = 3	0.5 dollar per Hourly use for applications
*k* = 1	1 dollar per Hourly use for applications Core I5 30 GB ROM
*k* = 2	2 dollar per Hourly use for applicationsCore I7 100 GB ROM
*k* = 3	3 dollar per Hourly use for applications Core I9 500 GB ROM

## Data Availability

All the experimental data are generated at the local institution servers. Therefore, it cannot be made publicly available for other researchers.
